# Supplementary material for: Cadmium Disrupted ER Ca2+ Homeostasis by Inhibiting SERCA2 Expression and Activity to Induce Apoptosis in Renal Proximal Tubular Cells
Source: Int J Mol Sci. 2023 Mar 22;24(6):5979. doi: 10.3390/ijms24065979 (PMC10053525; doi:10.3390/ijms24065979)
Supplement: Supplementary file 1 [file ijms-24-05979-s001.zip › ijms-2288173-supplementary.pdf]

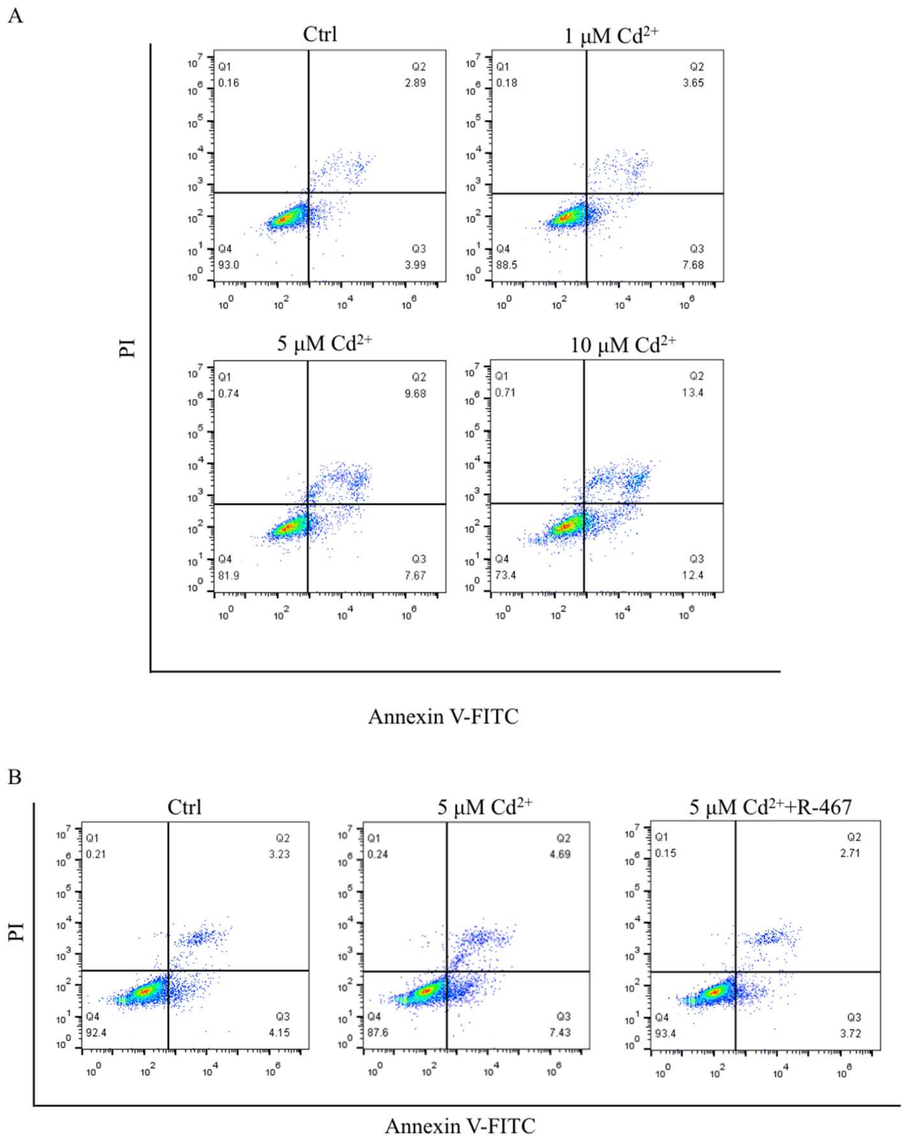

Figure S1. Effects of Cd2+ on apoptosis in mRTEC cells. After being treated with Cd2+ (1, 5, 10 μM) for 24 h, the apoptosis of mRTEC cells was determined by flow cytometry staining by Annexin V-FITC/PI.

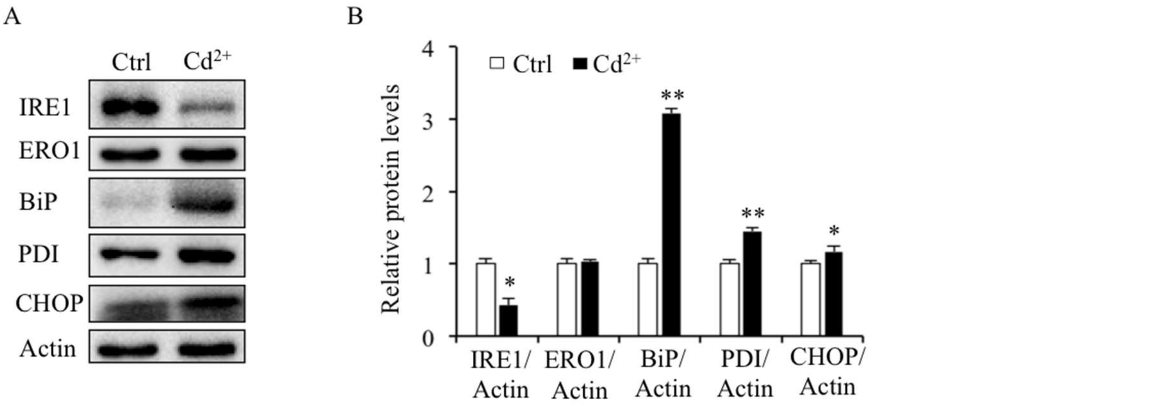

Figure S2. Effects of Cd^{2+} on ER stress. (**A**) Effect of Cd^{2+} on the expression of ER stress biomarkers IRE1, ERO1, BiP, PDI, and CHOP by Western blotting. (**B**) Quantification of the relative protein levels was performed using the software Image J. Statistical significance between control and treatments, *, $p < 0.05$, **, $p < 0.01$, using Student's t-test.
